# Geographical Patterns in Drug-Related Mortality and Suicide: Investigating Commonalities in English Small Areas

**DOI:** 10.3390/ijerph16101831

**Published:** 2019-05-23

**Authors:** Peter Congdon

**Affiliations:** School of Geography, Queen Mary University of London, Mile End Rd, London E1 4NS, UK; p.congdon@qmul.ac.uk

**Keywords:** drug-related deaths, suicides, small areas, deprivation, social fragmentation, rurality

## Abstract

There are increasing concerns regarding upward trends in drug-related deaths in a number of developed societies. In some countries, these have been paralleled by upward trends in suicide. Of frequent concern to public health policy are local variations in these outcomes, and the factors underlying them. In this paper, we consider the geographic pattern of drug-related deaths and suicide for 2012–2016 across 6791 small areas in England. The aim is to establish the extent of commonalities in area risk factors between the two outcomes, with a particular focus on impacts of deprivation, fragmentation and rurality.

## 1. Introduction

There are increasing concerns regarding upward trends in drug-related deaths in certain developed societies, including the US [1,2] and the countries constituting the UK, such as England and Scotland [3]. Almost a third of drug-related deaths in 2015 in Europe happened in the UK. Trends in suicide in countries experiencing growth in drug-related deaths are less consistent: suicide in the US has increased in the last decade [4], while in the UK there is a more stable pattern in suicide rates in recent years.

There are aetiological similarities between suicide and drug-related death, as harmful drug misuse may be considered a form of self-injurious behaviour [5]. Indeed, Case and Deaton [6] combine suicide, drug and alcohol misuse mortality, and cirrhosis as “deaths of despair”. For example, Al-Sharqi et al. [7] discuss commonalities in risk factors for self-injurious behaviour and substance abuse, “including coexisting mental and physical disorders, impulsivity, problem drinking, partner relationship problems, significant life stressors and events, previous suicide attempts, and lack of social and family support”.

There are also overlaps between these events from a point of view of diagnostic classification, such that Rockett et al. [8] proposed a combined category of self-injury mortality, including suicides and deaths from opioid and other drug self-intoxication. This reflects the fact that many suicides may be hidden among accidental drug-related deaths [9,10].

Of frequent concern to public health policy are local variations in these outcomes, and the factors underlying them. Much focus has been on individual risk factors for drug misuse and self-harming behaviour, but a full perspective involves consideration of community influences [11,12]. In the case of mortality, individual event data and individual risk factor profiles are usually not available, and analysis of spatial contrasts is therefore important. Spatial variations will in part reflect variations in the composition of populations, and ultimately the operation of risk factors at individual level (compositional influences). However, they may also reflect distinct place effects (contextual influences) [13,14].

The literature on spatial patterns in drug-related mortality, and the ecological (area level) influences) on such mortality, has focused especially on the influence of area socio-economic status [15,16], urban status [17], and area social cohesion [18]. Most published papers at small area level concern the US, though Griffiths et al. [8] considered drug-related deaths in England and Wales according to an urban settlement classification of electoral wards. Small area studies of suicide are more plentiful, including applications to UK, US and Australia [4,19,20].

In this paper, we consider the geographic pattern of drug-related deaths and of suicide, for the five year period 2012–2016. The data consist of all registered deaths from these causes across England, with 15,123 drug-related deaths (67.8% of the total is for males; 32.2% for females), and 23,517 suicides (76.3% of the total is for males, and 23.7% for females). They are based on usual residence at time of death, and may have been referred to a Coroner before registration; see Section 6.1 in [21]. The International Classification of Diseases (ICD) ranges for these data used are consistent with official definitions of drug-related deaths and of suicides, as in official reports by the UK Office for National Statistics [22,23]. The ICD ranges are specified in Appendix A, which includes a discussion of a definitional overlap between the two events due to drug-related suicides. This overlap is more relevant to females, since “violent and highly lethal methods such as firearm suicide and hanging are more frequent among men, whereas women often choose poisoning or drowning, which are less violent and less lethal.” [24]

The data were requested from the UK Office for National Statistics and are in fact online at https://www.ons.gov.uk/peoplepopulationandcommunity/birthsdeathsandmarriages/deaths/adhocs/undefinednumberofsuicidesanddrugpoisoningdeathsbysexandmiddlesuperoutputareasmsoasenglanddeathsregistered2012to2016. The geographic framework is provided by 6791 small areas in England, denoted as Middle Level Super Output Areas or MSOAs, which are nested within 326 Local Authorities. The aim is to establish the extent of commonalities in area risk factors between the two outcomes. The analysis focuses especially on the impacts of area deprivation, of area social fragmentation (as a proxy for neighbourhood social cohesion), and of area type (rural vs. highly urban). The model specification allows for spatially clustered but unobserved area risk factors [25].

The following sections outline the rationale for the risk factors applied to English small areas in the context of broader research evidence; consider simple bivariate associations between these risk factors and the two outcomes; and finally consider full regression models, the extent to which they show commonality in effects of risk factors, and the spatial pattern of excess relative risk. These models are fitted using the R-INLA package, which enables simplified estimation for Bayesian spatial regression [26].

## 2. Area Risk Factors for Drug-Related Deaths and Suicide

Many studies report that socio-economic deprivation and poverty are associated with higher drug misuse, drug-related mortality and increased suicide risks [27,28]. The impact of area deprivation is partly because it is an aggregate of individual risk factors for drug misuse and suicide, such as lower education [29], unemployment [30] and low income [31]. That is, area deprivation is partly a compositional measure of risk. However, it may also partly reflect contextual risks, or place effects per se. This shows in studies of mental illness, which, controlling for individual characteristics, show significant effects of area socioeconomic disadvantage [32,33]. The analysis below uses the UK government’s Index of Multiple Deprivation (or IMD) as a score measure for area socio-economic status.

Many studies have confirmed impacts on drug misuse and suicide of neighbourhood social cohesion, which is here represented inversely by an index of social fragmentation. Low cohesion (high fragmentation) is generally proxied by available indicators such as high residential turnover [24,31], high numbers of non-family households and one person households, above average numbers of non-married adults [27,34], and absence of community institutions promoting cohesion. Impacts of neighbourhood fragmentation/cohesion on the two outcomes are to some extent simply compositional; for example, living alone and being unmarried are risk factors for both suicide [35] and drug-related death [36]. Housing type and tenure mix may also be relevant: in the UK, private sector rented housing is a comparatively insecure, short-stay form of housing, with Swales and Tipping [37] reporting that privately renting tenants are less likely to feel they belong to their neighbourhood or trust neighbours. The social fragmentation score used here is obtained from principal component analysis of the four variables of Congdon [34] using 2011 Census data, which measure one person and non-married households, population turnover and private sector renting.

There are variations between countries in how far drug mortality and suicide are related to urbanity, or its converse rurality, and account may need to be taken of confounding influences (including access to means) and gender differences. In the US, Ho [38] noted a convergence in drug-related deaths between rural and urban areas, whereas a rural excess remains for suicide [4]. Regarding the UK, Gartner et al. [39] reported male suicide mortality to be 11 per cent higher in rural areas after adjustment for socio-economic deprivation in 2002–2004, and more contemporary evidence shows a continuing rural excess [40].

## 3. Bivariate Associations between the Outcomes and Area Risk Factors

Some preliminary impression of the associations between the two outcomes and the area risk factors can be obtained by considering bivariate associations. Here, these are represented by tabulations of drug-related deaths (DRD) and suicide according to deciles of the deprivation score and fragmentation scores, and for urbanity, a rural–urban classification based on the 2011 Census, or RUC11 classification for short [41]. The latter is an ordered 8-fold categorisation from most to least urban, with the extremes (1st and 8th categories) being “urban major conurbation” and “rural village and dispersed in a sparse setting”.

**Figure 1:**
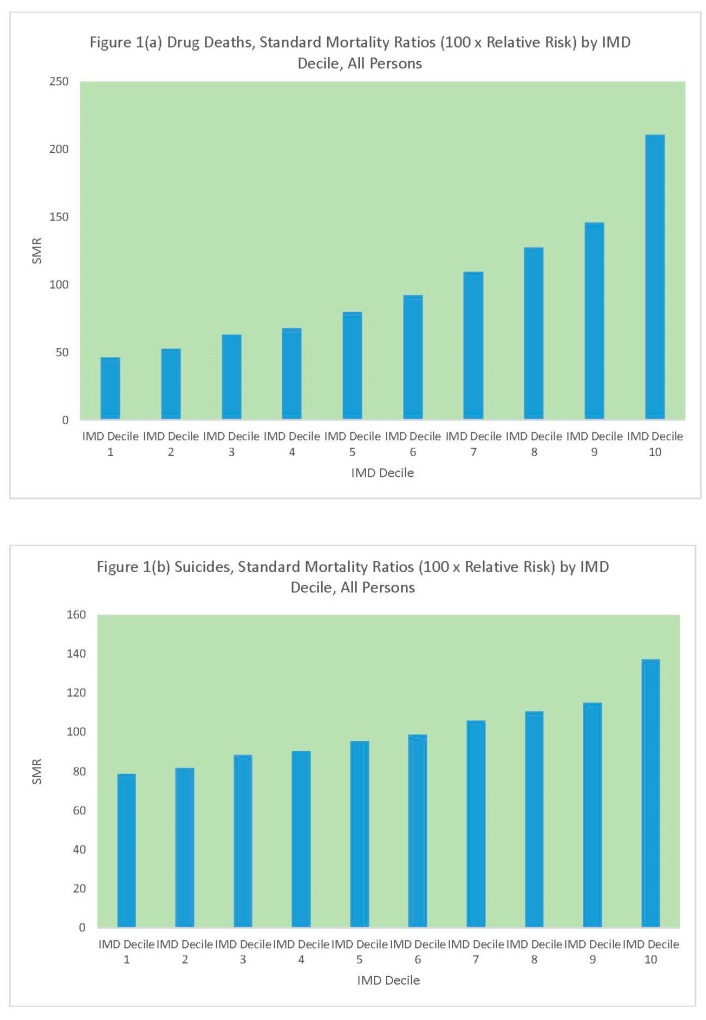
(**a**) DRD SMRs by MSOA Deprivation Decile, (**b**) Suicide SMRs by MSOA Deprivation Decile.

Table 1 and Figure 1 compare standard mortality ratios for drug-related deaths and suicides according to the deprivation (IMD) decile of the MSOA. It can be seen that there are regular upward gradients (increasing mortality with increased values of the risk factor) for both outcomes, but the upward gradient is sharper for drug-related deaths. The upward gradient in drug deaths is greater for males than females, with a 5.15 ratio in relative risk between the most and least deprived MSOAs for males.

**Figure 2:**
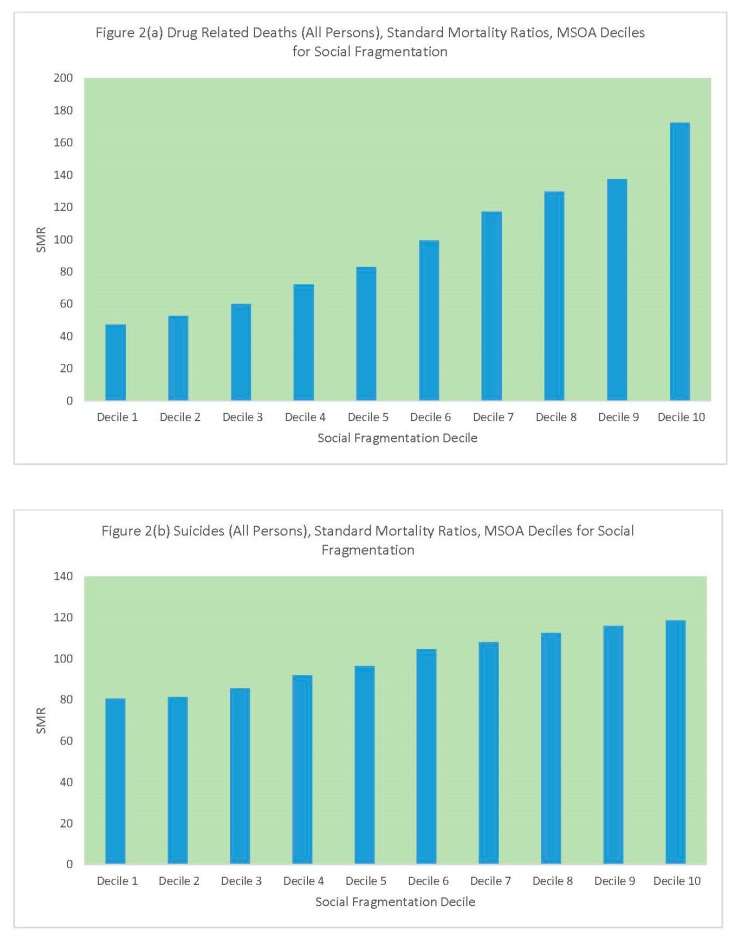
(**a**) DRD SMRs by MSOA Fragmentation Decile, (**b**) Suicide SMRs by MSOA Fragmentation Decile.

Similarly, Table 2 and Figure 2 compare standard mortality ratios for drug-related deaths and suicides according to the social fragmentation decile of the MSOA. Again, these are steeper for drug deaths than suicides, with a ratio of 4 in relative male DRD risk between the most and least fragmented MSOAs. Another feature of note is the stronger upward gradient in female suicide as compared to male suicide, as social fragmentation increases.

**Figure 3:**
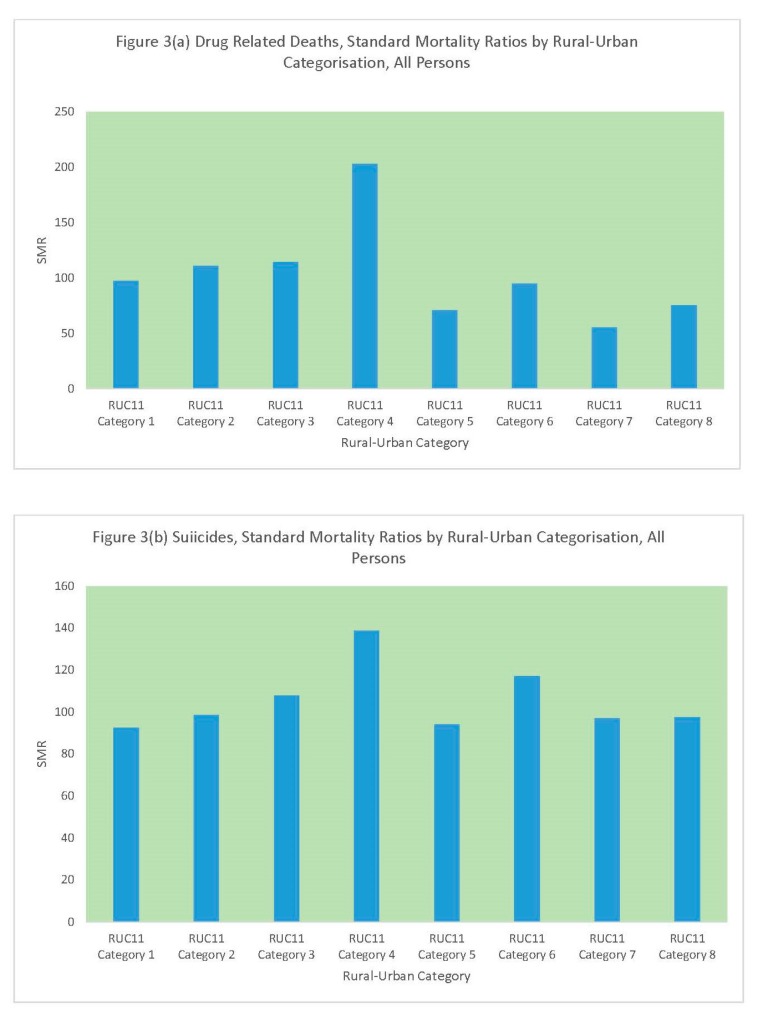
(**a**) DRD SMRs by MSOA Rural-Urban Category, (**b**) Suicide SMRs by MSOA Rural-Urban Category.

Finally, Table 3 and Figure 3 compare standard mortality ratios for drug-related deaths and suicides according to the rural–urban classification. The different categories contain differing numbers of MSOAs, with the category “urban city and town in a sparse setting” containing only 13 MSOAs. Hence, the elevated drug-related deaths standard mortality ratio for this category is representative of only a few small areas. Otherwise, the pattern for drug-related deaths seems to be of lower mortality in more rural categories, especially the categories “rural town and fringe”, “rural village and dispersed”, and “rural village and dispersed in a sparse setting”. By contrast, suicide mortality does not fall in these categories but is comparable to that in the three most urban categories: “urban major conurbation”, “urban minor conurbation”, and “urban city and town”.

These bivariate associations give some indication of what a more formal regression analysis would show, but do not allow for correlations between risk factors, or for the impacts of spatially correlated but unobserved risk factors. For example, there is a 0.46 correlation between deprivation and fragmentation, so their impacts may be attenuated in a full regression. The next section therefore considers findings from more formal regression, with technical details of the method contained in Appendix B.

## 4. Risk Factors and Relative Risks of Drug-Related Deaths and Suicides from Formal Regression

Here, we consider how far results from formal regression match the initial impressions gained from bivariate tabulations. The large number of areas in the study raises the chance of establishing significant effects of risk factors (with 95% intervals entirely positive or negative). Table 4 shows estimates of the regression coefficients (and 95% intervals for the coefficients) for each outcome and each risk factor. Also shown are the implied relative risks comparing neighbourhoods with extreme scores (highest and lowest scores). These can be obtained simply by exponentiating the coefficients, by virtue of the way covariates are expressed (see Appendix B): the highest deprivation and fragmentation scores have score 1 and the lowest have score 0. The extreme contrasts in drug deaths between highly deprived and highly affluent neighbourhoods shows in relative risks approaching 10 for males.

This Table shows that deprivation and fragmentation are significant positive risk factors for both drug-related deaths and suicides, but with the regression impacts stronger for drug deaths. The fragmentation effect is highly significant despite a positive correlation between fragmentation and deprivation. Rurality has no effect on drug deaths, but there is a significant positive impact on suicides (i.e., statistically positive, meaning increased suicide in rural areas), though the impact is not nearly as strong as that of deprivation and fragmentation.

To enable a more direct comparison with the preceding section, Table 5 and Table 6 show estimated standard mortality ratios according to the same categories used in Table 1, Table 2 and Table 3. Thus, Table 5 shows pronounced upward gradients in predicted drug deaths according to both deprivation and fragmentation. Drug-related mortality is 4.33 times more likely in the most deprived MSOAs than the least deprived, while such mortality is 3.55 times more likely in the most fragmented areas than the least. Deprivation and fragmentation tend to be lower in rural areas, and it can be seen that the categories rural town/fringe and rural village/dispersed have significantly lower drug-related deaths.

Table 6 also shows upward gradients in suicides according to both deprivation and fragmentation, albeit less pronounced than for drug deaths. For female suicides, the fragmentation gradient is steeper than that for deprivation, whereas the reverse is true for male suicides.

For suicides according to rural–urban category, possibly the most notable finding is significantly lower suicide in the most metropolitan settings (urban major conurbation), whereas the “urban city and town” categories have significantly elevated suicide mortality. Further analysis by broad English region (north, midlands, south) was undertaken (Appendix C). This shows the highest suicide SMRs to be in the “urban city and town” category in the North of England, including towns such as Blackpool, Middlesbrough, Barrow-in-Furness, Copeland and Preston, all of which have average predicted suicide SMRs (all persons) over 140.

Suicide risk in rural areas is around average in the two categories containing 94% of rural MSOAs (see Table 3), though the most sparse rural areas have excess suicide risk.

## 5. Concentrated Excess Risk

A notable feature of the distribution of excess risk at MSOA level, especially for drug-related deaths, is the marked concentration of excess risk. This has implications for targeted public health interventions.

**Figure 4:**
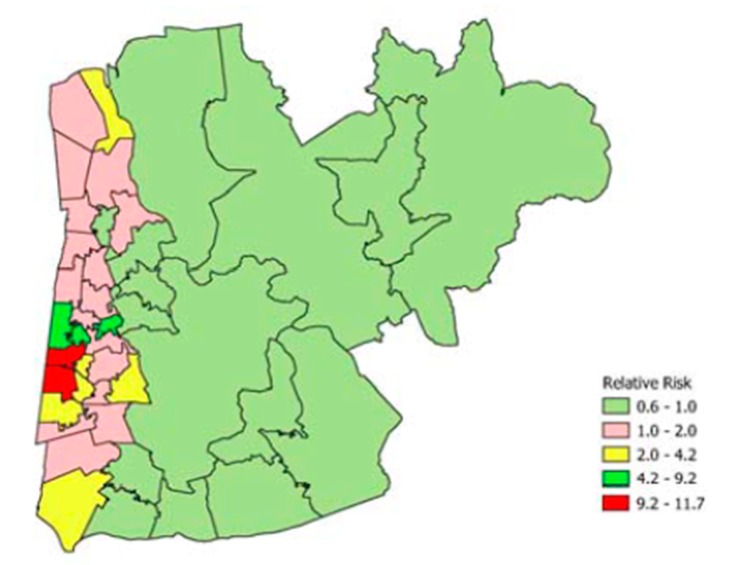
Drug-related death risk by MSOA (Blackpool, Fylde, Wyre).

Thus, Table 7 shows the 20 MSOAs with the highest predicted relative risk of drug-related death (all persons). It can be seen that five of the 20 are from one local authority, namely Blackpool. Map representation of Blackpool and two adjacent local authorities shows the clustering of extremely elevated DRD risk (Figure 4), with relative risks exceeding 10 in two MSOAs. The latter MSOAs are adjacent coastal MSOAs in Blackpool. The excess risk in these MSOAs contrasts with a background of lower than average risk in eastern MSOAs in the region mapped.

A distinguishing feature of the MSOAs in Table 7 is that they are all in the most deprived and fragmented deciles, confirming the strong relevance of these two risk factors to explaining DRD spatial contrasts.

**Figure 5:**
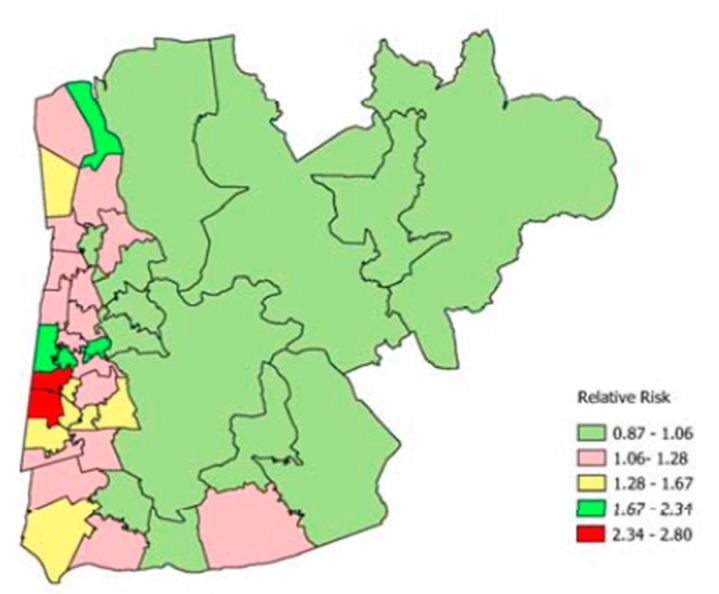
Suicide risk by MSOA (Blackpool, Fylde, Wyre).

As to suicide contrasts, the MSOAs with the highest predicted relative risks (all persons) are shown in Table 8. As for drug deaths, these MSOAs are also in the most fragmented and deprived deciles. However, the most extreme excess risks are less pronounced than for drug deaths, reaching only 2.8. Four of the highest suicide relative risks are in Blackpool, and comparison of Figure 4 and Figure 5 (for neighbourhoods in Blackpool and two adjacent local authorities) shows a spatial overlap in suicide and DRD excess risk.

In fact, across all 6791 English MSOAs, the correlation between DRD and suicide relative risks is 0.965. Interpreting this is subject to the caveat that the two events are not completely independent, since around a fifth of drug-related death figures are classified as suicides (method: drug overdose, or poisoning), and, conversely, a sixth of suicides are drug-related; see Appendix A [22,23]. However, the definitional overlap is unlikely to account for all this correlation.

## 6. Compositional vs. Contextual Effects

As for all health outcomes, a multilevel study considering area and individual risk factors in tandem may be regarded as the ideal, especially as there remains some debate about the existence of distinct area effects. In connection with that debate, one may say that many multilevel studies have a restricted geographic focus (e.g., on health variations within a city), not providing a proper comparative evaluation of area effects, and may not consider how contextual factors (e.g., changing local housing market opportunities) affect residential location of vulnerable groups at higher risk of (for example) drug-related deaths. Ecological (area-based) studies can have considerably greater coverage than multilevel studies. Thus, the present study is based on recent evidence comparing small areas across all of England and its nine regions.

Multilevel studies of psychiatric illnesses such as depression (which are risk factors for drug dependence and suicidality) have found contextual effects after control for individual risk factors. Thus, the authors of [33] mention “the daily stress of living in a neighborhood where residential mobility and material deprivation prevail is associated with depression [after control for individual attributes]”. Another study [42] finds a protective effect for low income people living in affluent areas. A multilevel study of suicide carried out by O’Reilly et al. [43] finds area effects to be absent after control for individual risk factors, but arguably provides only a relatively restricted intra-regional comparison (Northern Ireland), for an adult population of 1.6 million.

A broader comparison enables more complete evaluation of the impacts of local and regional labour markets, and of local and regional housing markets, which can be considered as primarily contextual factors. In that regard, the present study across enables a broader and more comprehensive assessment of relative risk differences across all nine English regions. It remains true that the present study is a population-based or ecological study [42], and “such studies cannot determine whether these are area effects (context) or due to the characteristics of the people living in these areas (composition)” [43].

However, the broad national scale of the present study means that some of the differences in relative risk identified may be plausibly linked to different housing and economic opportunities between and within regions. These may affect the residential location of at risk groups, and so reflect an interaction between contextual and individual risk factors.

For example, a recent House of Lords report on social and health problems in British coastal towns [44] mentions that “[a] decline in tourism, [has] left many seaside towns with a legacy of redundant tourist accommodation, including former hotels and bed and breakfast properties. Many of these properties [have been] converted to cater for the private rental market, leading to a dramatic growth in the number of HMOs [houses in multiple occupation] in seaside towns.” Such housing is often of low quality, attracting transient and vulnerable groups often living on state welfare support, and these changes adversely affect social cohesion. The operation of local housing factors in the rental market can be considered as a contextual influence, and its impact on the location of transient vulnerable groups may partly explain the extremely high rates of drug and suicide deaths in some MSOAs in Blackpool and other coastal towns. Thus, the highest relative risks for drug deaths (see Table 7 and Table 8) include an over-representation of neighbourhoods in coastal towns such as Blackpool, Clacton, Torbay, Hastings, Weymouth, Great Yarmouth and Brighton.

## 7. Conclusions

There are a number of small area studies of drug-related deaths and suicides. There have been more studies for both outcomes in the US, whereas in the UK there have been many more small area studies of suicide than of drug-related deaths.

A limitation of the present study is that it is ecological (area-based), so one cannot determine whether effects of area risk factors are due to context, or the characteristics of the people living in these areas (composition). However, as a counterbalance is the comprehensive coverage obtained of spatial variations in the two events, across all English small areas. The disaggregation of event counts by gender has shown gender differences in covariate effects. An additional strength is provided by a comparison of outcomes. No previous UK studies have compared the two outcomes for comparable time and spatial frameworks. The present study is therefore distinctive in carrying out a comparative small area study of the spatial patterning of drug deaths and suicides, which are two mortality types now often grouped under the umbrella category of “deaths of despair” [6,45]. A further strength is consideration of the impacts of deprivation and fragmentation in tandem: no previous small area study of UK drug deaths has considered social cohesion or fragmentation as a risk factor.

Future research on small area differences might consider outcome counts disaggregated by broad age group (e.g., young adults under 44 versus other age bands) as impacts of area risk factors may be age- as well as gender-differentiated. There is also scope to compare suicide, drug-related deaths and alcohol-related deaths, namely all components of deaths of despair.

As to findings of the present study, some contrasts between the outcomes are revealed: thus, drug-related deaths are distinct in showing extremely high relative risks in some MSOAs. However, the comparative analysis has shown commonalities, such as a considerable similarity in the spatial patterning of the two events. There is also strong similarity in the area risk factors for drug-related deaths and suicides, with particular regard to the impacts of deprivation and social fragmentation.

The impacts of these two risk factors are significantly positive for both outcomes, but the impacts are stronger for drug deaths. Whereas a number of existing studies have shown social fragmentation to be a significant positive risk factor for suicide, the present paper is the first showing that there is a strong impact of social fragmentation on drug-related deaths also.

Of relevance to interpreting these contrasts are the misclassification issue mentioned in the Introduction (nominally accidental drug deaths are in fact suicide), and also the outcome definitional overlap (see Appendix A). It has been suggested that the potential for misclassification is greater among women [46], since “the less immediately lethal methods of suicide preferred by women, such as self-poisoning, are more likely to be mis-classified as accidental than the more immediately lethal methods of suicide preferred by men”.

Thus, the deprivation and fragmentation effects identified by regression in the paper are stronger for drug-related deaths than for suicides, as officially defined. However, to the extent that drug-related deaths are concealed suicides, it may be that the deprivation and fragmentation effects for suicides are understated, and this understatement is more likely for females. It could also be suggested that the reason that drug-related deaths may show a stronger correlation with deprivation, compared to suicide deaths, is due to social stigma, making drug misuse deaths more prevalent or visible in more deprived areas. It might also be suggested that stronger deprivation and fragmentation effects for drug-related deaths reflect higher levels of illicit drug misuse in more deprived areas, as opposed to prescription drug misuse.

Gender differences in impacts of area covariates are apparent from the regressions, and raise potential questions regarding the relative impact of ‘gendered’ risk factors. Thus, for female suicides, the fragmentation gradient is steeper than that for deprivation, whereas the reverse is true for male suicides. Such findings are consistent with differential gender risk factor effects: for example, loss of employment may be more strongly related to masculine despair, whereas loss of social support and lower social integration are more strongly related to feminine despair [47,48,49]. Thus, the authors of [50] mention that “the relationship between unemployment and suicide, and socio-economic status and suicide, appears to be stronger for men than it is for women. Overall, then, men are more sensitive to negative changes in their socio-economic and employment status, and this may lead to higher risks for suicide”.

Implications for public health intervention of the findings here may be considered. Thus, highly localised excess risks of both events, but especially drug-related deaths, point to the need for local neighbourhood targeting of interventions. The HM Government’s drug strategy [51] mentions “a targeted approach for high priority groups”, and “targeting the most vulnerable”, and this approach may need to take into account how housing or labour market factors lead to concentration of vulnerable groups in particular neighbourhoods. Similarly, the findings here support targeted suicide interventions, in line with [52], which proposes that interventions “should recognise the strong association between suicidal behaviour and area-level socioeconomic deprivation, targeting efforts on both people and places.” Deciding which areas have highest priority for prevention measures is often based on socioeconomic scoring procedures, and the findings here point to the need to consider social fragmentation (as an inverse index of social cohesion) as well as area deprivation.

## Figures and Tables

**Table 1:** Drug-related deaths (DRD) and suicides, standard mortality ratios by Middle Level Super Output Area (MSOA) deprivation decile.

MSOA IMD Decile	Persons		Males		Females	
	DRD	Suicides	DRD	Suicides	DRD	Suicides
IMD Decile 1	46	79	42	75	55	89
IMD Decile 2	53	82	50	80	59	86
IMD Decile 3	63	88	57	85	76	99
IMD Decile 4	68	90	70	92	64	84
IMD Decile 5	80	95	78	94	82	101
IMD Decile 6	92	99	91	100	94	95
IMD Decile 7	109	106	108	107	112	102
IMD Decile 8	128	110	128	110	125	112
IMD Decile 9	146	115	147	118	143	104
IMD Decile 10	210	137	216	139	197	132

**Table 2:** Drug-related deaths (DRD) and suicides, standard mortality ratios by MSOA fragmentation decile.

MSOA SFI Decile	Persons		Males		Females	
	DRD	Suicides	DRD	Suicides	DRD	Suicides
SFI Decile 1	47	80	43	81	57	79
SFI Decile 2	53	81	50	81	58	81
SFI Decile 3	60	86	59	85	63	87
SFI Decile 4	72	92	71	92	76	92
SFI Decile 5	83	96	80	98	90	91
SFI Decile 6	100	105	98	106	104	99
SFI Decile 7	117	108	120	109	114	103
SFI Decile 8	130	112	130	112	130	113
SFI Decile 9	138	116	134	116	143	117
SFI Decile 10	173	119	172	114	158	136

**Table 3:** Drug-related deaths (DRD) and suicides, standard mortality ratios by rural–urban categorization (England Standard Mortality Ratio (SMR) = 100).

RUC11 Category	Number of MSOAs	Persons		Males		Females	
		DRD	Suicides	DRD	Suicides	DRD	Suicides
Urban major conurbation	2399	97	92	96	93	99	90
Urban minor conurbation	249	111	99	112	100	106	94
Urban city and town	2938	114	108	115	107	113	110
Urban city and town in a sparse setting	13	203	139	217	151	179	101
Rural town and fringe	588	71	94	70	94	74	95
Rural town and fringe in a sparse setting	20	95	117	88	126	108	89
Rural village and dispersed	539	55	97	54	97	59	95
Rural village and dispersed in a sparse setting	45	75	98	77	97	73	99

**Table 4:** Estimated Regression Coefficients (Drug Related Deaths and Suicides).

		Mean	2.5%	97.5%	Relative Risk Comparing Areas with Extreme Scores *
DRD	Persons				
	Deprivation	2.13	2.00	2.25	8.41
	Fragmentation	1.78	1.62	1.94	5.93
	Rurality	−0.09	−0.22	0.05	0.91
	Males				
	Deprivation	2.26	2.12	2.41	9.58
	Fragmentation	1.71	1.53	1.90	5.53
	Rurality	−0.02	−0.19	0.14	0.98
	Females				
	Deprivation	1.82	1.63	2.01	6.17
	Fragmentation	1.53	1.27	1.78	4.62
	Rurality	0.04	−0.15	0.23	1.04
Suicides	Persons				
	Deprivation	0.90	0.80	1.00	2.46
	Fragmentation	0.68	0.55	0.81	1.97
	Rurality	0.19	0.1	0.27	1.21
	Males				
	Deprivation	0.99	0.88	1.10	2.69
	Fragmentation	0.53	0.39	0.68	1.70
	Rurality	0.21	0.11	0.30	1.23
	Females				
	Deprivation	0.55	0.35	0.74	1.73
	Fragmentation	1.14	0.89	1.38	3.13
	Rurality	0.24	0.09	0.40	1.27

* Relative Risk, Neighbourhood with Maximum Score vs Neighbourhood with Minimum Score.

**Table 5:** Estimated Standard Mortality Ratios, Drug Related Deaths *.

Deprivation	Persons	Males	Females
IMD Decile 1	**51.2**	**48.8**	**57.0**
IMD Decile 2	**57.9**	**55.7**	**64.0**
IMD Decile 3	**63.0**	**60.6**	**68.8**
IMD Decile 4	**69.8**	**68.0**	**75.6**
IMD Decile 5	**78.5**	**76.6**	**82.5**
IMD Decile 6	**89.4**	**87.4**	**91.8**
IMD Decile 7	**104.6**	**102.3**	**105.4**
IMD Decile 8	**119.8**	**118.6**	**118.2**
IMD Decile 9	**143.4**	**143.9**	**139.4**
IMD Decile 10	**222.0**	**225.8**	**211.3**
Fragmentation	Persons	Males	Females
SFI Decile 1	**51.1**	**49.0**	**57.9**
SFI Decile 2	**60.9**	**59.4**	**67.3**
SFI Decile 3	**65.8**	**64.5**	**71.7**
SFI Decile 4	**74.4**	**73.4**	**79.1**
SFI Decile 5	**84.1**	**83.1**	**87.6**
SFI Decile 6	98.8	98.0	100.7
SFI Decile 7	**116.2**	**116.5**	**114.9**
SFI Decile 8	**129.9**	**129.8**	**127.5**
SFI Decile 9	**137.0**	**135.1**	**134.5**
SFI Decile 10	**181.4**	**178.8**	**172.8**
Urban-Rural Category **	Persons	Males	Females
Urban major conurbation	100.0	**98.5**	**104.1**
Urban minor conurbation	**114.0**	**115.8**	**109.3**
Urban city/town	**110.5**	**108.8**	**108.2**
Urban city/town (sparse)	**165.6**	**166.7**	**144.4**
Rural town/fringe	**72.7**	**72.5**	**78.3**
Rural town/fringe (sparse)	98.5	98.5	106.7
Rural village/dispersed	**65.0**	**64.9**	**72.9**
Rural village/dispersed (sparse)	93.5	95.2	99.7

* Significantly Elevated or Depressed SMRs in Bold, ** For full category names see Table 3.

**Table 6:** Estimated Standard Mortality Ratios, Suicides*.

Deprivation	Persons	Males	Females
IMD Decile 1	**78.2**	**77.0**	**82.0**
IMD Decile 2	**83.9**	**83.1**	**86.9**
IMD Decile 3	**87.9**	**87.0**	**91.0**
IMD Decile 4	**91.6**	**90.9**	**94.3**
IMD Decile 5	**94.7**	**94.0**	**97.3**
IMD Decile 6	**98.8**	**98.4**	100.1
IMD Decile 7	**103.9**	**103.6**	**105.1**
IMD Decile 8	**108.3**	**108.4**	**107.9**
IMD Decile 9	**116.4**	**118.0**	**111.7**
IMD Decile 10	**138.4**	**142.2**	**126.6**
Fragmentation	Persons	Males	Females
SFI Decile 1	**80.5**	**80.9**	**79.4**
SFI Decile 2	**86.6**	**87.1**	**85.8**
SFI Decile 3	**89.7**	**90.0**	**89.2**
SFI Decile 4	**93.6**	**94.2**	**91.9**
SFI Decile 5	**96.8**	**97.7**	**94.3**
SFI Decile 6	**102.4**	**103.1**	99.4
SFI Decile 7	**108.2**	**109.2**	**104.5**
SFI Decile 8	**111.7**	**112.3**	**108.9**
SFI Decile 9	**110.7**	**109.9**	**113.0**
SFI Decile 10	**121.8**	**117.9**	**136.6**
Urban-Rural Category **	Persons	Males	Females
Urban major conurbation	**93.7**	**94.4**	**92.5**
Urban minor conurbation	**100.6**	**103.6**	**92.2**
Urban city/town	**105.8**	**105.0**	**107.3**
Urban city/town (sparse)	**133.4**	**134.9**	**126.3**
Rural town/fringe	**97.9**	**98.2**	98.9
Rural town/fringe (sparse)	**113.3**	**113.7**	**117.3**
Rural village/dispersed	**98.1**	**98.4**	99.4
Rural village/dispersed (sparse)	**115.6**	**116.8**	**117.8**

* Significantly Elevated or Depressed SMRs in Bold, ** For full category names see Table 3.

**Table 7:** MSOAs with Highest DRD Relative Risk, With Socio-Economic and Locational Details.

			Relative Risk (95% Interval)		
MSOA Name	Local Authority	Region	Mean	2.5%	97.5%
Blackpool 010	Blackpool	North West	11.67	8.60	15.31
Blackpool 013	Blackpool	North West	11.26	7.68	15.61
Blackpool 006	Blackpool	North West	9.22	6.36	12.72
Tendring 016	Tendring	East of England	7.71	4.95	11.20
Blackpool 008	Blackpool	North West	7.57	5.15	10.56
Wirral 016	Wirral	North West	6.23	4.47	8.39
Thanet 001	Thanet	South East	6.15	4.04	8.75
Great Yarmouth 006	Great Yarmouth	East of England	5.88	3.94	8.28
Sunderland 016	Sunderland	North East	5.73	3.83	8.10
Blackpool 007	Blackpool	North West	5.67	3.67	8.25
Shepway 014	Shepway	South East	5.64	3.40	8.52
Middlesbrough 003	Middlesbrough	North East	5.43	3.66	7.63
Sefton 004	Sefton	North West	5.43	3.75	7.52
Stockton-on-Tees 014	Stockton-on-Tees	North East	5.26	3.94	6.84
Hastings 011	Hastings	South East	5.23	3.41	7.52
Plymouth 029	Plymouth	South West	5.18	3.59	7.13
Barrow-in-Furness 008	Barrow-in-Furness	North West	5.17	3.24	7.67
Weymouth and Portland 004	Weymouth and Portland	South West	5.00	3.25	7.20
Waveney 007	Waveney	East of England	4.93	3.24	7.07
Burnley 007	Burnley	North West	4.84	3.09	7.14

**Table 8:** MSOAs with Highest Suicide Relative Risk, With Socio-Economic and Locational Details.

MSOA Name	Local Authority	Region	Mean	2.5%	97.5%
Blackpool 013	Blackpool	North West	2.80	2.02	3.76
Thanet 001	Thanet	South East	2.76	1.96	3.74
Tendring 016	Tendring	East of England	2.74	1.93	3.75
Blackpool 010	Blackpool	North West	2.71	2.09	3.46
Torbay 008	Torbay	South West	2.48	1.84	3.26
Cornwall 068	Cornwall	South West	2.43	1.56	3.55
Hastings 011	Hastings	South East	2.42	1.75	3.24
Middlesbrough 003	Middlesbrough	North East	2.36	1.79	3.06
Blackpool 006	Blackpool	North West	2.34	1.73	3.08
Great Yarmouth 006	Great Yarmouth	East of England	2.33	1.71	3.09
Barrow-in-Furness 008	Barrow-in-Furness	North West	2.30	1.63	3.14
Blackpool 008	Blackpool	North West	2.23	1.65	2.93
Middlesbrough 002	Middlesbrough	North East	2.19	1.68	2.80
Kingston upon Hull 024	Kingston upon Hull	Yorkshire & Humberside	2.17	1.70	2.73
Shepway 014	Shepway	South East	2.17	1.42	3.14
Sefton 004	Sefton	North West	2.15	1.66	2.75
Stockton-on-Tees 014	Stockton-on-Tees	North East	2.14	1.75	2.60
Middlesbrough 001	Middlesbrough	North East	2.13	1.75	2.56
Lancaster 009	Lancaster	North West	2.10	1.49	2.86
Brighton and Hove 030	Brighton and Hove	South East	2.09	1.56	2.73

## Appendix A. Definition of Outcomes by ICD and Overlap Due to Drug-Related Suicides

Deaths related to drugs are for underlying causes defined by International Classification of Diseases, Ninth and Tenth Revisions (ICD-9 and ICD-10) codes:

**Description**	**ICD-9 Codes**	**ICD-10 Codes**
Mental and behavioural disorders due to drug use (excluding alcohol and tobacco)	292, 304, 305.2–305.9	F11–F16, F18–F19
Accidental poisoning by drugs, medicaments and biological substances	E850–E858	X40–X44
Intentional self-poisoning by drugs, medicaments and biological substances	E950.0–E950.5	X60–X64
Assault by drugs, medicaments and biological substances	E962.0	X85
Poisoning by drugs, medicaments and biological substances, undetermined intent	E980.0–E980.5	Y10–Y14

Deaths due to suicide are defined as deaths with an underlying cause of intentional self-harm or injury/poisoning of undetermined intent, with ICD-10 codes:

**ICD-10 Codes**	**Description**
X60-X84	Intentional self-harm
Y10-Y34	Injury/poisoning of undetermined intent

There is an overlap between the outcomes. The overlap arises from intentional self-poisoning by drugs, medicaments and biological substances (ICD X60–X64), and poisoning by drugs, medicaments and biological substances, undetermined intent (Y10–Y14); these are an element in both suicides and drug deaths. Thus, for the years 2015–2017, and across all England, there were 13,846 suicides, of which 2316 (16.7%) were drug-related suicides (ICD10 X60–X64, Y10–Y14); the percentage drug-related is higher for females (31.4%) than for males (11.8%). In the same period, there were 10,348 drug-related deaths, of which 22.4% were drug-related suicides; the percentage is higher for females (33%) than males (17.4%).

## Appendix B. Regression Methods

The method used in this paper is widely adopted in applications usually described as Bayesian disease mapping [53]. Specifically, we used a form of generalised linear model to estimate relative risks for the two outcomes, with the model incorporating an assumption (a prior belief) that there is likely to be correlation between risk in areas geographically close. For both outcomes, the response is a count variable and the outcome is relatively rare, so a Poisson log-linear model is assumed based on known risk factors X (e.g., deprivation) and including a spatially structured error term. So for counts Y_i_ of a particular outcome in area i (for example, drug deaths) we have

Y_i_ ~Poisson(E_i_ρ_i_),

log(ρ_i_) = X_i_β+ν_i_,

where E_i_ are expected drug deaths, ρ_i_ is the relative risk of DRD for area i, β are regression coefficients for known risk factors X_i_, and ν_i_ is a random effect representing unexplained spatial correlation. These random effects are assumed to follow a conditional autoregressive distribution, as developed by Besag et al. [54], with the spatial effect for area i depending on spatial effects in adjacent areas. Also following Besag et al. [54], we include a spatially independent random effect u_i_ to represent geographically unstructured heterogeneity in disease risk. Then the log-linear model for relative risk is

log(ρ_i_) = X_i_β+ν_i_+u_i_.

Estimation of the models is carried out using the R-INLA package. In the regression, we represent the risk factors IMD and social fragmentation in (0,1) form, so that the highest deprivation and fragmentation scores have score 1 and the lowest have score 0. Putting risk factors on the same scale in this way means the relative importance of their impacts can be assessed. To represent urban–rural status, a ridit score of rurality is obtained based on the ordered 2011 rural/urban categories [55]. This score is in (0,1) form by definition, with 1 representing the most rural areas. This scoring approach is adopted to avoid potentially unstable estimates if a categorical predictor were used instead, since some categories have small numbers of MSOAs in them.

## Appendix C. Grouping of Regions

We grouped the nine standard regions of England into three broader groupings as follows:
